# Screening and Evaluation of Xanthine Oxidase Inhibitors from *Gnetum parvifolium* in China

**DOI:** 10.3390/molecules24142671

**Published:** 2019-07-23

**Authors:** Xiaosheng Tang, Ping Tang, Lei Ma, Liangliang Liu

**Affiliations:** 1 Hubei Key Laboratory of Edible Wild Plants Conservation and Utilization & National Demonstration Center for Experimental Biology Education & College of Life Sciences, Hubei Normal University, Huangshi 435002, China; 2 Zhengzhou Research Base, State Key Laboratory of Cotton Biology, Zhengzhou University, Zhengzhou 450001, China; 3 School of Environmental Science and Engineering, Hubei Polytechnic University, Hubei Key Laboratory of Mine Environmental Pollution Control and Remediation, Huangshi 435003, China; 4 Institute of Bast Fiber Crops, Chinese Academy of Agricultural Sciences, Changsha 410205, China

**Keywords:** antioxidants, *Gnetum parvifolium*, inhibitor screening, ultrafiltration, xanthine oxidase

## Abstract

As a traditional natural medicine for treating many kinds of diseases, *Gnetum parvifolium* showed apparent inhibition on xanthine oxidase (XO). In this study, ultrafiltration combined with liquid chromatography-mass spectrometry (LC-MS) is used for the screening of XO inhibitors from *Gnetum parvifolium*. Their antioxidation, XO inhibition, and enzymic kinetic parameters are also determined. Finally, piceatannol (**1**), rhaponiticin (**2**), resveratrol (**3**), and isorhapontigenin (**4**) are screened out and identified as XO inhibitors from the extract of *Gnetum parvifolium*. Four inhibitors show better inhibition than allopurinol and good radical scavenging abilities. However, the antioxidant activities are weaker than ascorbic acid. The kinetic parameters illustrate the inhibition mode of XO by piceatannol is competitive type, while the inhibition modes for rhaponiticin, resveratrol and isorhapontigenin are uncompetitive types. In order to evaluate the difference among samples obtained in China, the amounts of four inhibitors and related activities in 20 samples are assessed and analyzed by partial least squares analysis. The results indicate piceatannol contribute the highest coefficients in three kinds of activities. Based on these findings, more comprehensive research on pharmaceutical and biochemical activities of these four XO inhibitors could be conducted in future.

## 1. Introduction

*Gnetum parvifolium* is a green liana species belonging to the genus *Gnetum*. Its stem and root are usually used as natural medicines with acceptable therapeutic effects in treating rheumatic, aching limbs, acute respiratory infections, chronic bronchitis, and traumatic injuries in China [1,2]. Previous chemical research reported that flavonoids, stilbenoids, and alkaloids are several of the main components in *Gnetum parvifolium* [3]. Some findings showed the extract of *Gnetum parvifolium* could effectively inhibited the activity of xanthine oxidase (XO) in vitro and reduce the level of blood uric acid in mice (Reference in Chinese). However, the report on particularly active components related to this activity is rare.

Plenty of active compounds exist in natural products. It makes natural products an invaluable source for exploring pharmaceutical and chemical active compounds [4]. However, various kinds of components make the finding, identification, and separation of active compounds complicated and time-consuming [5]. Among many novel screening methods, ultrafiltration combined with liquid chromatography-mass spectrometry (LC-MS) is an easy and efficient screening strategy based on specific protein-ligand interaction [6]. Ultrafiltration combined with LC-MS has become a useful tool for screening and identifying affinity ligands of proteins and enzymes from natural products [7,8,9]. During the ultrafiltration process, enzyme and bound ligands are trapped by membrane and further analyzed by LC-MS. Unnecessary isolation of inactive compounds is avoid in this screening method. Because this method focuses on target binding activity, it promotes the efficiency of analysis and simplifies the processes of experiment [10].

In this study, the inhibition of *Gnetum parvifolium* extract on XO in vitro is confirmed in our lab. XO inhibitors in *Gnetum parvifolium* extract are screened and identified using ultrafiltration combined with LC-MS. Four compounds are identified under the optimum screening conditions. Based on these results, detailed inhibition and antioxidant activities of these four compounds were conducted. In order to evaluate the difference among samples botained in China, 20 *Gnetum parvifolium* samples distributed in four provinces were collected and compared as well.

## 2. Results and Discussion

### 2.1. Screening and Identification of XO Ligands

Before the screening experiment, the inhibition of *Gnetum parvifolium* extract on XO was tested in order to guarantee if the further screening and evaluation of inhibitors would be worthwhile. As a result, the ethanol extract of *Gnetum parvifolium* showed apparent inhibition with IC_50_ value of 58.5 μg/mL, which indicated that the *Gnetum parvifolium* extract contained XO inhibitors.

**Figure 1:**
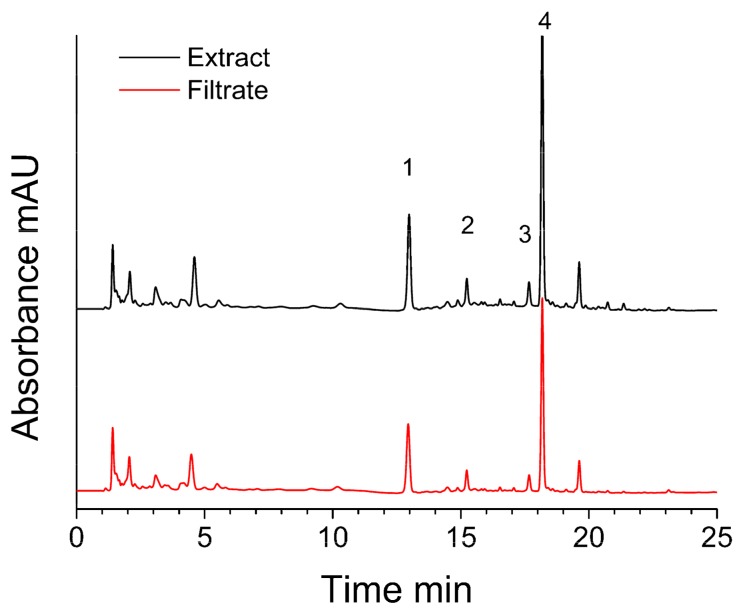
The chromatogram of *Gnetum parvifolium* extract (black) and filtrate after ultrafiltration (red).

During ultrafiltration processes, ligands from extract bind to enzyme and form enzyme-ligand complexes in incubation. The complexes would remain during ultrafiltration by membrane. Meanwhile, the unbound compounds would pass through the membrane. Accordingly, in the chromatographic analysis, the peak areas of ligands would decrease after ultrafiltration [11]. Therefore, the decrease of peak area becomes an indicator for evaluation. After the optimization of chromatographic conditions, the representative chromatogram of *Gnetum parvifolium* extract and that of filtrate are shown in Figure 1. About four peaks could be considered as the ligands because the peak areas of them are decreased in filtrate. The validations of method are conducted by screening using positive and negative controls. The screening with denatured enzyme is also carried out. The results showed the screening is specific (data not shown).

**Figure 2:**
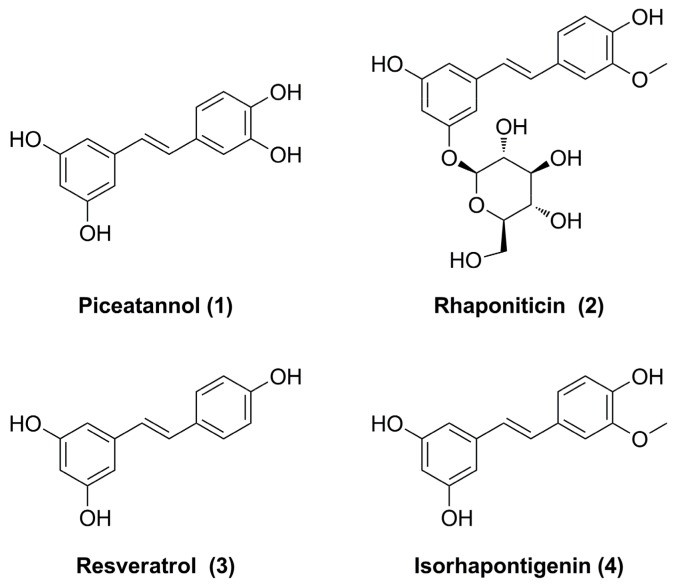
The chemical structures of four inhibitors from *Gnetum parvifolium* extract.

High-performance liquid chromatography-mass spectrometry (HPLC-MS) analysis is then used to identify the marked four ligands. The structures of them are identified by analyzing and comparing their retention times, UV data, and MS data with those of authentic samples. The UV spectra of four compounds show wide absorbance at around 310 nm. This is the typical spectra of stilbenoids, which are abundant in *Gnetum parvifolium*. In negative mode, the MS spectra of four compounds show the deprotonated molecular ions ([M − H]^−^) at 243, 419, 227, and 257, respectively. According to the previous reported information of stilbenoids, the retention times and MS spectra of authentic references are selected and compared with those of extract sample. Finally, they are identified as piceatannol (**1**) [12], rhaponiticin (**2**) [13], resveratrol (**3**) [14], and isorhapontigenin (**4**) [15] (Figure 2 and Table 1).

### 2.2. Inhibition and Kinetics Studies of Four Inhibitors

The inhibition of four ligands on XO is tested in vitro. As a result, the four ligands show good inhibitions with IC_50_ values of 6.44, 5.997, 3.8,8 and 46.75 μM for piceatannol (**1**), rhaponiticin (**2**), resveratrol (**3**), and isorhapontigenin (**4**), respectively. Resveratrol shows the highest inhibitory activity, followed by rhaponiticin, piceatannol, and isorhapontigenin. As a commercially obtained inhibitor for XO, allopurinol is used as a positive control with the IC_50_ value of 52.0 μM. Therefore, these four ligands could be considered as satisfying inhibitors for XO. Schmeda-Hirschmann et al. reported the methanol extract of *Scirpus californicus* inhibited XO, of which piceatannol was one of the targeted isolated components [16]. Gaballah et al. reported that resveratrol could markedly suppress XO and increase glutathione peroxidase activity [17]. However, the inhibitions on XO of rhaponiticin and isorhapontigenin were not previously reported.

**Figure 3:**
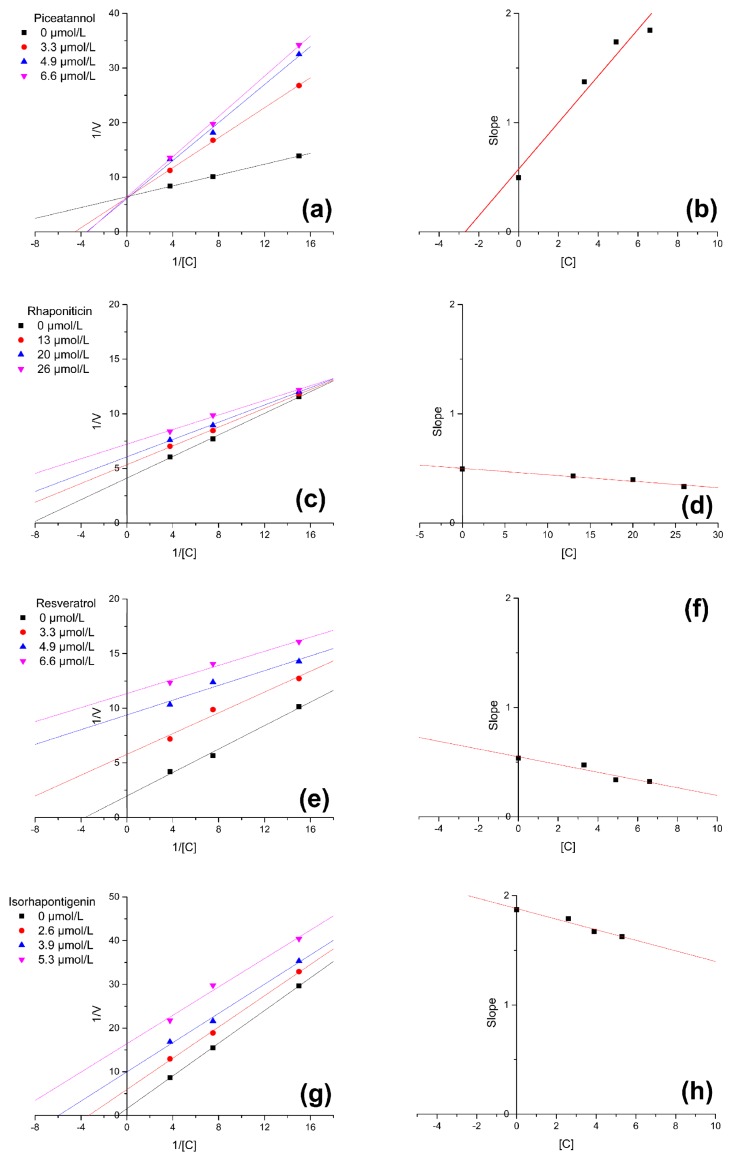
Lineweaver-Burk plots for inhibition of xanthine oxidase (XO) by (**a**) piceatannol, (**c**) rhaponiticin, (**e**) resveratrol, and (**g**) isorhapontigenin. Secondary plots of slopes against concentrations of (**b**) piceatannol, (**d**) rhaponiticin, (**f**) resveratrol, and (**h**) isorhapontigenin to calculate *K_i_*.

In order to further exploring the inhibitory activities of four inhibitors, the inhibition type and inhibition kinetics constants were assayed. The activity was tested with different concentrations of substrates (0.067, 0.133, and 0.267 mM) and different concentrations of inhibitors (3.3, 4.9, and 6.6 μM for piceatannol and resveratrol; 13, 20, and 26 μM for rhaponiticin; 2.6, 3.9, and 5.3 μM for isorhapontigenin). Figure 3 shows the Lineweaver-Burk plots for piceatannol, rhaponiticin, resveratrol, and isorhapontigenin and Table 2 shows some parameters. It could be seen through the plots that the mode of XO inhibition by piceatannol is competitive type and the modes of XO inhibition by rhaponiticin, resveratrol, and isorhapontigenin are uncompetitive type. The competitive inhibition means the Michaelis-Menten constant (*K_m_*) was increasing and the maximum reaction velocity (*V_max_*) remained unchanged, which indicates the occupation by inhibitor prevents substrate from connecting to the active site of enzyme [18]. The uncompetitive inhibition means both *K_m_* and *V_max_* are decreased, which indicates the inhibitor caused the inhibition by forming enzyme-substrate complex reversibly with weak interactions at a site other than the active site [19]. The *K_i_* values derived from secondary plots are 0.0027, 0.085, 0.052, and 0.039 mM for piceatannol, rhaponiticin, resveratrol, and isorhapontigenin, respectively. Smaller value of inhibition constant indicates stronger inhibition and the inhibitor-enzyme binding affinity exceeded the enzyme-substrate binding affinity [20].

### 2.3. Antioxidant Activity

Antioxidant activities of four inhibitors were also determined by DPPH free radical scavenging and ABTS radical scavenging methods. The radical scavenging results are presented as EC_50_ values in Table 2. Ascorbic acid was used as the positive control with IC_50_ values of 0.02 mM for DPPH and 0.02 mM for ABTS. The rank in scavenging activities from the highest to the lowest is piceatannol, rhaponiticin, resveratrol, and isorhapontigenin for both DPPH and ABTS. The results show these four inhibitors have antioxidant activities as well. However, the scavenging activities of them are weaker than that of ascorbic acid. Lee et al. reported that piceatannol and resveratrol could scavenge DPPH free radical [21]. Yang et al. also reported the radical scavenging activities of piceatannol and isorhapontigenin [22]. However, the radical scavenging activities of rhaponiticin is first reported in this study.

### 2.4. Comparison of Inhibitors among Different Gnetum Parvifolium Samples

In order to calculate the contents of these four inhibitors in various *Gnetum parvifolium* distributed in China, 20 *Gnetum parvifolium* samples from four provinces were obtained and analyzed (detail information was shown in Table S1). The contents of four inhibitors, antioxidant activities, and XO inhibition are evaluated and shown in Table 3. Sample 13 showed the maximum activities for DPPH, ABTS, and XO, while sample 16 showed the minimum activities for DPPH and ABTS and the second minimum for XO. The partial least squares analyses among four active compounds and activities were performed. As shown in Table 4, piceatannol contributes the highest coefficients for investigated three activities. Therefore, it could assume that the content of piceatannol in *Gnetum parvifolium* affects the investigating activities mostly.

## 3. Materials and Methods

### 3.1. Chemicals and Materials

Xanthine oxidase (XO) freeze-dried powder was purchased from Yuanye Biotechnology Co. (Shanghai, China). Acetic acid and Acetonitrile in HPLC grade were purchased from Merck KGaA (Darmstadt, Germany). 1,1-Diphenyl-2-picrylhydrazyl (DPPH) and 2,2′-azinobis-(3-ethylbenzthiazoline-6-sulfonic acid (ABTS) were bought from Merck KGaA (Darmstadt, Germany). Ultrapure water (18.2 MΩ cm resistivity) was obtained from an ELGA water purification system (ELGA Berkefeld, Veolia, Germany). Piceatannol (96.0%) and resveratrol (99.0%) were commercially acquired from Acros Organics (Fisher Scientific, PA, USA). Rhaponiticin (96.0%) and Isorhapontigenin (96.0%) were purchased from Yuanye Biotechnology Co. (Shanghai, China). All other chemicals were analytical grade and purchased from Sinopharm Chemical Reagent Co., Ltd. (Shanghai, China). Twenty *Gnetum parvifolium* samples were bought from pharmacies in Anhui, Guangxi, Hebei, and Guangdong province.

### 3.2. Extraction of Gnetum Parvifolium

*Gnetum parvifolium* was extracted by microwave-assisted extraction method [23]. Twenty grams of samples were transferred into a conical flask containing 200 mL of 90% ethanol solution. The solution was extracted for 4 min in microwave oven at 60% power (800 W, 2450 MHz, Galanz, G80W23CSL-A6, Guangdong, China). After extraction three times, the solvents were combined and evaporated with rotary evaporator under vacuum. Finally, 2.04 g of residues were dissolved in methanol or water and stored at 4 °C for further use.

### 3.3. Inhibition Assay on XO

For inhibition test, 20 μL of XO solution (1 mg/mL in buffers) and 1 mL of sample with different concentrations were mixed in a quartz cuvette. Then, 1 mL of XO (1 mg/mL) was added to start reaction and the increase of absorbance at 295 nm in 100 s was recorded by an UV-Vis Spectrophotometer (UV2700, Shimadzu, Kyoto, Japan) [24]. Allopurinol was used as a positive control and water was used as a blank. The inhibitions of ethanol and methanol on XO were conducted, and there was no inhibition on XO for these two solvents. All experiments were performed in triplicate. The inhibition of XO was calculated by the following formula.

Inhibition% = (1 − ΔA_s_/ΔA_0_) × 100 (1)

where ΔA_s_ and ΔA_0_ are the changes of absorbance for sample and blank, respectively. The inhibition of sample was expressed as the concentration of sample needed to inhibit 50% of enzymatic activity (IC_50_).

### 3.4. Screening of XO Inhibitors Using Ultrafiltration

The screening was conducted using ultrafiltration according to our previous reports with some modifications [25]. Two-hundred microliters of XO solution (1.0 mg/mL) and 200 μL of *Gnetum parvifolium* extract (100 mg/mL) were mixed in a tube and shaken at 25 °C for 60 min with a thermostatic oscillator. After incubation, the mixture was transferred into a centrifugal filter (YM-30, the molecular weight cut off of 10 kDa) and centrifuged at 15492.5× *g* for 20 min at 4 °C (Beckman Coulter Allegra 64R, Brea, CA, USA). The filtrate was collected for HPLC-MS analysis and the enzyme along with binding inhibitors were retained by membrane. Four-hundred microliters of buffer solution were added into the filter and the filter was centrifuged at the same conditions for the second time. Then, the filtrate was decanted and another 200 μL of 80% methanol solution were added into the filter. The filter was centrifuged at 15492.5× *g* for 20 min at 4 °C to elute binding inhibitors from enzymes and the filtrate named as eluent was collected for HPLC-MS. The control experiment was carried out in the same condition with denatured enzyme after high-temperature processing as a substitution.

### 3.5. HPLC-MS Analysis

The qualitative and quantitative analysis of screening was achieved by HPLC-MS analysis on an Agilent 1260 HPLC combined Agilent 6460 Triple Quadrupole LC-MS system (Agilent Technologies Inc., Santa Clara, CA, USA). The HPLC separation was completed using a C_18_ reverse phase column (Thermo Fisher Scientific, Hypersil GOLD, 100 mm × 2.1 mm i.d., 3 μm, Waltham, MA, USA). A gradient elution program consisting of water and acetonitrile was used as follows: 0–5 min, 10% acetonitrile and 5–35 min, 10%–35% acetonitrile. The flow rate was set at 0.2 mL/min and the column temperature was set at 25 °C. The chromatogram was recorded at 254 nm. For mass spectrometry analysis, the Electron Spray Ionization (ESI) interface was used in negative ionization mode. The mass detection mode was set at full-scan mode from 100 to 1000 *m*/*z*.

### 3.6. Antioxidation Tests

The DPPH and ABTS· radical scavenging tests were measured according to the reported method with some modifications [26,27]. For DPPH test, 0.5 mL of sample with different concentrations were mixed thoroughly with 2 mL of DPPH solution (0.1 mM in methanol). The mixture was then kept in dark for 30 min. Finally, the absorbance of solution was measured by an UV-Vis Spectrophotometer (UV2700, Shimadzu, Kyoto, Japan) at 517 nm.

For ABTS test, ABTS solution (2 mL of ABTS stock solution (0.01 M) added to 58 mL phosphate buffers, pH 7.0) was incubated for 12 h and diluted with methanol for use. Then, 0.5 mL of samples with different concentrations were mixed thoroughly with 2 mL of ABTS solution. The mixture was kept in dark for 30 min, and the absorbance at 734 nm was measured by an UV-Vis Spectrophotometer. The same amount of water was used as control instead of sample. All experiments were performed in triplicate. The scavenging activity of DPPH and ABTS· radicals was calculated by the following formula:

Radical scavenging rate% = 1 − A_s_/A_0_ × 100 (2)

where A_0_ was the absorbance of control and A_s_ was the absorbance of sample. The radical scavenging activity of sample was expressed as the concentration of sample needed to scavenge 50% of DPPH or ABTS (IC_50_).

## 4. Conclusions

In current study, ultrafiltration combined with HPLC-MS was used to screen XO inhibitors from *Gnetum parvifolium*. Piceatannol (**1**), rhaponiticin (**2**), resveratrol (**3**), and isorhapontigenin (**4**) were finally screened out and identified. They show good inhibitions and acceptable antioxidant activities compared with positive control. The inhibitions on XO of rhaponiticin and isorhapontigenin, as well as the radical scavenging activities of rhaponiticin, are first reported. The kinetic parameters exhibited the mode of XO inhibition by piceatannol is competitive type, while the inhibition modes for rhaponiticin, resveratrol, and isorhapontigenin are uncompetitive type. The amounts of four inhibitors and related activities in twenty samples obtained in China are assessed. The findings could contribute to a comprehensive understanding of the pharmaceutical potential use of *Gnetum parvifolium* as a natural source for XO inhibitors. These results also provide more phytochemical information for active constituents in *Gnetum parvifolium.*

## Figures and Tables

**Table 1:** Identification of ligands in *Gnetum parvifolium* by liquid chromatography-mass spectrometry (LC-MS).

Peak	Rt (min)	UV (nm)	[M − H]^−^	Mw	Formula	Identification
1	13.0	220, 325	243	244	C_14_H_12_O_4_	Piceatannol
2	15.2	220, 330	419	420	C_21_H_24_O_9_	Rhaponiticin
3	17.7	215, 305	227	228	C_14_H_12_O_3_	Resveratrol
4	18.2	220, 325	257	258	C_15_H_14_O_4_	Isorhapontigenin

**Table 2:** EC_50_ values, IC_50_ values, and kinetic parameters in enzymatic reactions of four inhibitors.

Compounds	DPPH EC_50_ (mM)	ABTS EC_50_ (mM)	XO IC_50_ (μM)	[C] (μM)	*K_m_* (mM)	*V_max_* (mmol/min)	*K_i_* (mM)
Piceatannol	0.04	0.01	6.44	0	0.077	0.155	0.0027
				3.3	0.220	0.160	
				4.9	0.287	0.164	
				6.6	0.290	0.157	
Rhaponiticin	0.13	0.04	5.97	0	0.120	0.243	0.085
				13	0.081	0.187	
				20	0.065	0.165	
				26	0.046	0.139	
Resveratrol	1.34	0.23	3.88	0	0.273	0.508	0.052
				3.3	0.082	0.173	
				4.9	0.036	0.106	
				6.6	0.028	0.088	
Isorhapontigenin	1.45	0.30	46.75	0	0.450	0.281	0.039
				2.6	0.301	0.168	
				3.9	0.249	0.134	
				5.3	0.104	0.061	

**Table 3:** The contents of four inhibitors, radical scavenging activities and XO inhibition of twenty *Gnetum parvifolium* samples. The concentration of each extract was unified at 2.0 mg/mL.

No.	Contents (mg/g)				Bioactivity (Inhibition %)		
	Piceatannol	Rhaponiticin	Resveratrol	Isorhapontigenin	DPPH	ABTS	XO
1	1.7	60.1	37.4	184.0	33.5	69.2	36.4
2	4.1	47.5	65.0	238.1	34.9	72.3	26.9
3	5.7	51.2	71.6	169.8	40.2	76.8	83.8
4	12.4	28.8	26.8	89.0	34.1	75.2	93.0
5	0.8	36.0	40.2	201.1	42.2	76.7	66.2
6	2.7	19.9	31.2	112.6	30.1	70.6	64.9
7	2.5	12.0	32.5	36.6	355	83.3	93.7
8	1.7	26.8	19.5	114.5	3.5	78.2	80.9
9	3.4	14.0	22.2	61.7	3.0	68.7	55.7
10	6.6	36.1	53.5	163.9	36.8	72.6	80.4
11	10.6	78.4	62.7	272.1	36.8	70.7	40.3
12	3.2	23.8	16.8	289.2	55.4	80.5	65.3
13	16.6	56.1	39.5	153.9	77.8	96.0	96.2
14	11.3	175.3	31.6	514.2	33.6	71.5	39.8
15	9.8	65.2	57.6	271.7	50.6	80.2	77.1
16	3.2	12.5	25.8	248.0	28.6	62.6	27.6
17	5.4	41.0	52.5	304.8	37.4	78.7	89.4
18	5.1	34.5	15.9	202.1	63.2	92.7	92.8
19	1.1	18.1	20.3	164.3	46.2	90.7	47.7
20	1.8	53.5	19.5	240.1	69.3	88.6	74.8

**Table 4:** The parameters of partial least squares analysis of four active compounds and activities.

Compounds	Partial Least Squares Analysis		
	DPPH	ABTS	XO
Piceatannol	6.1743	2.8243	10.5320
Rhaponiticin	−4.2698	−1.2723	−4.4226
Resveratrol	−4.1071	−2.6934	−2.4919
Isorhapontigenin	3.4288	−0.2928	−7.6642

## Supplementary Materials

The following are available online. Table S1: The locations, batch numbers and species of twenty Gnetum parvifolium samples.
