# Supplementary material for: Screening and Evaluation of Xanthine Oxidase Inhibitors from Gnetum parvifolium in China
Source: Molecules. 2019 Jul 23;24(14):2671. doi: 10.3390/molecules24142671 (PMC6680845; doi:10.3390/molecules24142671)
Supplement: Supplementary file 1 [file molecules-24-02671-s001.pdf]

Table S1 The locations, batch numbers and Species of twenty *Gnetum parvifolium* samples.

No.	Location	Batch No.	Species
1	Guangxi	83256	*Gnetum parvifolium*
2	Hebei	70927	*Gnetum parvifolium*
3	Guangxi	05784	*Gnetum parvifolium*
4	Guangxi	85557	*Gnetum parvifolium*
5	Guangdong	14190	*Gnetum parvifolium*
6	Anhui	83364	*Gnetum parvifolium*
7	Guangdong	75939	*Gnetum parvifolium*
8	Guangxi	98243	*Gnetum parvifolium*
9	Anhui	95301	*Gnetum parvifolium*
10	Guangxi	11709	*Gnetum parvifolium*
11	Anhui	14190	*Gnetum parvifolium*
12	Guangxi	23549	*Gnetum parvifolium*
13	Anhui	24108	*Gnetum parvifolium*
14	Hebei	53556	*Gnetum parvifolium*
15	Anhui	62717	*Gnetum parvifolium*
16	Guangxi	20848	*Gnetum parvifolium*
17	Anhui	19393	*Gnetum parvifolium*
18	Anhui	51882	*Gnetum parvifolium*
19	Anhui	66161	*Gnetum parvifolium*
20	Anhui	79801	*Gnetum parvifolium*
